# Health shocks, basic medical insurance and common prosperity: Based on the analysis of rural middle-aged and elderly groups

**DOI:** 10.3389/fpubh.2022.1014351

**Published:** 2022-12-09

**Authors:** Yuan Zhang, Yuquan Sun, Mingli Xie, Yuping Chen, Shouhui Cao

**Affiliations:** ^1^School of Business Administration, Zhongnan University of Economics and Law, Wuhan, China; ^2^Food, Agriculture and Resource Economics, University of Guelph, Guelph, ON, Canada

**Keywords:** health shock, aging population, common prosperity, basic medical insurance, China

## Abstract

Health is a major part of human welfare. The index system of common prosperity was constructed for middle-aged and elderly people in rural areas. Besides, the impart of health shocks and rural basic medical insurance on common prosperity was explored. The data for this study came from China Health and Retirement Longitudinal Survey (CHARLS) in 2013, 2015, and 2018. The finding shows that health shocks hindered the improvement of the common prosperity of the middle-aged and elderly in rural areas, among which daily activities produced the greatest negative effect. The heterogeneity analysis shows that health shocks have a stronger negative effect on the common prosperity of low-income groups than that of high-income ones. The shock of daily activity ability has the greatest influence on the middle-aged and elderly between 45 and 55 years old. However, acute health shocks have a strong negative effect on those aged above 56. The mechanism analysis shows that rural basic medical insurance can alleviate the health shocks to middle-aged and elderly people, but the effect is limited. In general, low-income groups benefit more. Therefore, China should speed up the promotion of the Healthy China Strategy and the reform of the rural basic medical insurance system, and prompt changes from an inclusive to a targeted policy to provide more precise safeguards for vulnerable groups.

## Introduction

Aging is one of the major trends in the changing age structure of the population in the world, and it is a main economic and social issue against the development of all nations (1). China is also so. According to the prediction of the World Bank, the population of the elderly in China will exceed 400 million by 2,035, with an aging rate of about 28%, and most of them live in rural areas (2). Unlike other groups, elderly people are highly vulnerable to health shocks due to their weak social roles and poor physical conditions, especially given the relative lack of family support and medical care facilities in rural China. Statistics show that around 66% of the elderly aged above 60 in rural areas suffer from chronic diseases, which is much higher than that of the elderly in urban areas. This requires China to face a challenge in the long term: the health of the rural elderly.

As clearly pointed out by the Fifth Plenary Session of the 19th Central Committee, “the common prosperity of all people will have achieved more obvious and substantial progress by 2,035”. On par with the new concept of common prosperity by the World Bank, the concept of common prosperity is aimed at enabling poor people to benefit from economic growth (3). Common prosperity lays emphasis on social equity and justice and comprehensive and coordinated development. Health, an indispensable human capital, is an important factor in enhancing the earning power of individuals and the wellbeing of humans (4). Safeguarding the health of rural elderly people is an important part of achieving comprehensive health and an important step to realize common prosperity. This is because the health problems of elderly people in rural areas are related to their development and life quality, as well as the harmonious development of the entire family. They are also closely associated with the wellbeing of the whole society. On the one hand, health shocks reduce the labor supply of middle-aged and elderly people and other family members by generating unexpected medical expenditures, leading to a decline in household income and increased uncertainty about household income (5–15). On the other hand, from a social perspective, the physical health status of middle-aged and elderly people, especially the elderly, is directly related to the care burden and medical expenses of the whole society (16, 17).

The main way for families to deal with health shocks is to increase preventive savings and participate in medical insurance, and the latter is a more effective means of risk transfer (18). At present, rural areas have established a full coverage medical insurance and security system to guarantee that each individual has medical insurance. Through social welfare, the material and spiritual living conditions of rural residents have been continuously improved, enabling them to share the achievement of economic and social development. From the existing literature, some scholars believe that rural medical insurance helps to improve the physical and mental health of participants (19–23), reduce household medical expenditures and preventive savings (24, 25), and enhance the ability to cope with health shocks (26). Nonetheless, some scholars stated that rural basic medical insurance has little effect on the improvement of residents' health and economic welfare (27, 28). Despite the increase in the utilization of medical services by patients, the rapid growth of medical expenses has resulted in no decline in their costs (29), which partially impairs the protection function of medical insurance (30). The economic and health effects of medical insurance have been extensively explored in the above literature, but most of them use data from a single year or non-tracking surveys and focus on the average coverage function of medical insurance for various groups of people. In a rapidly aging society, we must focus on the elderly people and explore the function of rural medical insurance for the elderly. This will contribute to the reform of rural medical insurance system and further improve its welfare effects.

This study aims to analyze the impact of health shocks on the common prosperity of the rural middle-aged and elderly, and verify the effects of rural basic medical insurance in the face of health shocks. The data for this study came from China Health and Retirement Longitudinal Survey (CHARLS) in 2013, 2015, and 2018. Compared with existing research, this study made the following contributions. This study firstly focused on middle-aged and elderly people in rural areas, and then it builds a multi-dimensional coupled common prosperity index system for them. In addition, the people-oriented concept of common prosperity was comprehensively reflected. Second, multi-dimensional health indicators were selected. The impacts of different health shocks on the common prosperity of the middle-aged and elderly in rural areas were subdivided, finding that daily activities have a greater negative effect than other health shocks. Third, whether rural basic medical insurance can alleviate the negative impact of health shocks on the common prosperity of the middle-aged and elderly in rural areas was explored. Additionally, the beneficiary groups of rural basic medical insurance were further evaluated.

## Data, variables, and empirical model

### Data

The 2013, 2015, and 2018 three-phase tracking data of CHARLS were used, aiming to collect a nationally representative micro-data set of the Chinese population aged 45 and above to analyze the problem of Chinese population aging. The national baseline survey began in 2011, whose content covers personal basic information, health status, household income and consumption, medical insurance, etc. to meet the research needs of the middle-aged and elderly. This paper dealt with the data set as follows: Firstly, the samples whose household registration is agricultural were surveyed in 2013, 2015, and 2018, and retained; secondly, the samples with missing key information including individuals and families were eliminated, and the final balanced panel data is 20,256.

### Variables

#### Dependent variable

Level of common prosperity (CP). Common prosperity is the continuation and expansion of poverty alleviation work (31). It emphasizes social fairness and justice and the all around development of people, including material prosperity, spiritual life and sharing of social development achievements. It refers to the prosperity of all the people, which requires benefiting each individual. Rural middle-aged and elderly groups account for about 49.38% of the total rural population, which thus cannot be ignored in the process of promoting common prosperity. Previous studies focused on the development of middle-aged and elderly people in rural areas from material and spiritual dimensions (32, 33), embodying the “prosperity” dimension of common prosperity. The focus of common prosperity should be on “prosperity” and “common” dimensions. It should incarnate the fairness and equality of human development, narrow the urban-rural gap between middle-aged and elderly people in terms of income and living standards, and eliminate their insufficient access to medical resources. The disparity in basic public services is also an important part of promoting common prosperity. According to previous studies, an index evaluation system was constructed for the common prosperity of middle-aged and elderly people in rural areas from the three dimensions of material and spiritual wealth as well as social sharing. Subdivision indicators for dimensions including livable pension environment and active aging spirit were selected (see Table 1).

**Table 1:** Common prosperity index system for middle-aged and elderly people in rural areas.

**First-level** **indicators**	**Second-level** **indicators**	**Third-level** **indicators**	**Critical value**
Material wealth	Income	Absolute income	If the per capita annual income of the family is not less than CNY2300, the value is 1; otherwise, 0
		Relative income	If the per capita annual household income is not lower than the national median disposable income of rural residents in that year, the value is 1; otherwise, 0
		Subjective relative income	Compared with the average living standard of the village/neighbor, if you think your living standard is similar or above, assign a value of 1; otherwise,0
	Employment	whether have a job	If the respondent is a business farmer or a worker, the assignment is 1; otherwise, 0
	Consumption	Per capita consumption	If the total per capita consumption is not lower than the rural per capita consumption of the province in that year, and the value is 1; otherwise, 0.
	Living environment	Tap water	Yes = 1, no = 0
		Internet	Yes = 1, no = 0
		Cooking fuel	Clean fuel = 1, no = 0
		Whether the toilet can be flushed	Yes = 1, no = 0
		Housing structure	Reinforced concrete or brick and wood structure, the value is 1; otherwise, 0
		Cleanliness of the house	Neat, assign a value of 1; otherwise, 0
Spiritual wealth	Health	Self-assessed health	The self-assessment of good health is assigned a value of 1; Otherwise, 0
	Education	Education level	1 for junior high school and above; otherwise, 0
		Vocational training	Whether to participate in vocational training, yes = 1, no = 0
	Social security	Endowment insurance	Whether to participate in or receive any pension insurance, yes = 1, no = 0
	Culture	Social interaction	Whether you have participated in social activities in the past month, yes = 1, no = 0
		Subjective wellbeing	Evaluation of life satisfaction, satisfaction = 1, otherwise 0
Social sharing	Group differences	Objective difference	If the ratio of local urban and rural income does not exceed the sample median of the year, assigned a value of 1; otherwise, 0
		Subjective difference	Compared with the average living standard of people in this county/city/district, if you feel that your living standard is about the same or above, assign a value of 1; otherwise, 0
	public service provision	Medical resources per capita	If the number of health institutions per 10,000 people is not lower than the sample median of the year, assigned a value of 1; otherwise, 0
	Regional differences	The level of economic development	If the region is the east, the value is 1; otherwise, 0

Referring to the previous literature (32–34), the dimension of material wealth is mainly measured from income, wealth, consumption and living environment. It mainly highlighted that a secure life for the middle-aged and elderly, a livable environment and other basic survival needs were met. The income included both indicators reflecting objective income (absolute and relative income) and subjective indicators reflecting the income gap. The absolute poverty line of CNY2300 per year (at 2010 prices; this is approximately USD361) will be used as set by the Chinese government, which was assigned a value of 1, and otherwise 0. For relative income, the median disposable income of rural residents in the country in the current year was selected as the critical value. The per capita annual income of households in the current year was not lower than the critical value, whose value was assigned as 1 and otherwise 0. The relative poverty line of the 3 years can be calculated as CNY 7,907(USD1,276.23), CNY10,291 (USD1, 652.27) and CNY13,066(USD1,976.16).^1^ Regarding subjective relative income, the question on the measurement of relative income in the questionnaire “Do you think your standard of living is much better, better, similar, worse or much worse than the average living standard of your neighbors/village?” was used. It was assigned a value of 1 if the respondent's answer was similar, better or much better, and otherwise 0. Employment included agricultural and non-agricultural employment as long as the respondent was engaged in any of the two, and it was assigned a value of 1 and otherwise 0. The threshold value of consumption adopted the rural per capita consumption in the province where the respondent was located. It was assigned a value of 1 and otherwise 0 when the total per capita consumption of the household in the current year was not lower than the rural per capita consumption of the province in the current year. Each subdivision indicator was limited by space and would not be repeated one by one. Details are shown in Table 1.

Different from material wealth, spiritual wealth aims to reflect people's learning and development, protection of rights and interests as well as other aspects of a better life at a deeper level. The measure of the mental affluence dimension is difficult to define. The measurement of spiritual wealth mainly included culture, education and entertainment, political gain, etc. (1), while that of spiritual wealth included health, social security, education and culture (34). Limited by the availability of data, the latter definition was mainly drawn on. Health adopted self-assessment health, and good and above were assigned a value of 1 and otherwise 0. Education covered educational level and vocational training. The critical value of educational level was junior high school, and the value of junior high school and above was 1 and otherwise 0. For rural middle-aged and elderly people, labor participation is still an important basis for their survival and development (15), thereby suggesting that participation in vocational training is also crucial for their further improvement of human capital. Social security was measured by whether to participate in or receive any pension insurance. The cultural dimension covered social activities and subjective wellbeing. Social activities were represented by “Have you performed the following social activities in the past month” in the questionnaire, and the value was 1 if the respondent participated in any of them, and otherwise 0.

The social sharing dimension included group and regional differences into the social sharing dimension and covered the subdivision dimension of public service supply differences (34). Group differences included objective and subjective differences. Objective differences meant that the ratio of local urban-rural income did not exceed the sample median of the year as the critical value. However, subjective differences were based on the question on the measurement of relative income in the questionnaire “What do you think you are compared with the average living standard of people in this county/city/district? Is the standard of living much better, better, about the same, worse or much worse?” It was assigned a value of 1 when the respondent answered about the same or above, and otherwise 0. The supply of public services mainly reflected local medical resources. The median of the current year was selected as the critical value. When the number of local health institutions per 10,000 people was not lower than the median of the current year, the value was 1 and otherwise 0. Regional differences mainly considered the level of economic development. The sample region is the east, which was assigned a value of 1 and otherwise 0.

In addition, equal and entropy weight methods, analytic hierarchy processes, etc. were primarily used for determining weights. Among them, the equal weight method has been recognized and widely used by numerous scholars at the level of micro-individual data (34, 35), which was thus employed to assign indicators. Notably, subjective relative income and subjective differences in subdivision indicators were only included in 2013, and vocational training indicators were only reflected in 2015 and 2018 because of data availability.

#### Independent variable

Health shock is widely defined as sudden health deterioration caused by diseases or accidents (11, 36). Some previous studies used self-reported health (37), sickness in the past 4 weeks (37) and hospitalization in the past year (38), the proportion of medical expenditures (39) and other indicators as standards. However, the measurement of these indicators did not subdivide the types of health shocks. This tends to exaggerate the effect of health shocks and leads to the difficulty in truly and objectively reflecting the true impact of health shocks on individuals. Zhang et al. (40) stated that health shocks could also be segmented into chronic (*Sick_1*) and acute health (*Sick_2*) as well as daily activity ability shocks (*ADLS*) utilized in this study. Among them, chronic and acute health shocks were defined by “Whether you have been told by a doctor that you have a certain disease” in the database. As classified by Yang et al. (14, 36), the samples with heart disease, cancer or stroke were defined as acute health shocks and assigned a value of 1 and otherwise. In addition, chronic health shocks were defined as high blood pressure, stomach disease, rheumatoid arthritis, etc., and characterized according to the number of common chronic diseases the respondents suffered from. Middle-aged and elderly people were classified as acute health shock groups if suffering from two types of diseases simultaneously.

As regards the impact of daily activity ability, the questionnaire DB010-DB020 on 11 daily life behaviors such as dressing, bathing, eating, getting up and out of bed, and going to the toilet was used. Scores were summed up, with a value ranging from 11 to 44. The larger the value was, the greater the impact of its daily activity ability would be. The number of chronic diseases and the scores of daily activities of the respondents were standardized according to the following formula:


(1) $$\begin{array}{l} \hat{X}=\frac{X-\min\left(X\right)}{\max\left(X\right)-\min\left(X\right)} \end{array}$$

#### Control variables

Control variables were mainly selected from personal and family characteristics according to previous literature (36, 37, 39). The level of personal characteristics included: (1) The age of the respondents (*age*) was a continuous variable; (2) Marital status (*marry*) was a binary variable. The value of marriage was 1, and that of divorce and widowhood was 0; (3) Whether it is a party member (*party*) was a binary variable; (4) Type of residence (*live*). Family residence was assigned a value of 1, and a nursing home or other elderly care institution was assigned a value of 0.The level of family characteristics covered: (5) Family size (*hsize*) was a continuous variable; (6) The existence of productive fixed assets (*invest*) was a binary variable, the family has productive fixed assets such as threshers, tractors, etc., was assigned a value of 1 and otherwise 0; (7) Whether to take care of grandchildren (*care*), two categorical variables; (8) per capita annual household income (*income*), a continuous variable, incorporating the logarithm of income into the regression equation.

#### Mediating variable

The role of basic medical insurance between health shocks and common prosperity among middle-aged and elderly people was explored in this study. Some areas in China have made a combination of new rural cooperative medical insurance and urban resident medical insurance collectively referred to as urban and rural resident medical insurance. Therefore, the value was 1 and otherwise 0 if the respondents participating in new rural cooperative medical care or basic medical insurance were considered to participate in basic medical insurance in rural areas. Additionally, individuals participating in both basic medical insurance and other types of medical insurance, including commercial medical insurance, urban employee medical insurance, etc. were deleted for better testing of the role of rural basic medical insurance.

The above variables and their descriptive statistics are shown in Table 2.

**Table 2:** Descriptive statistics.

**Variables**	**Symbols**	**2013**		**2015**		**2018**	
		**Mean**	**S.D**.	**Mean**	**S.D**.	**Mean**	**S.D**.
**Dependent variable**							
Common prosperity for the middle-aged and elderly in rural areas	CP	0.4964	0.1429	0.5248	0.1294	0.5530	0.1367
**Independent variable**							
Activities of daily living shock	ALDS	0.0321	0.0836	0.0421	0.0974	0.0527	0.1126
Chronic health shock	Sick_1	0.1287	0.1886	0.1721	0.2153	0.2506	0.2523
Acute health shock	Sick_2	0.1401	0.3471	0.1881	0.3908	0.2922	0.4548
**Control variables**							
Age	Age	58.8520	8.4786	60.8520	8.4786	63.8520	8.4786
Marital status	Marry	0.9159	0.2776	0.9159	0.2776	0.9274	0.2595
Party member	Party	0.9285	0.2577	0.9285	0.2577	0.9362	0.2445
Type of residence	Live	0.9954	0.0676	0.9852	0.1208	0.9767	0.1507
Household population size	Hsize	3.5579	1.8617	2.5255	1.1743	2.7656	1.4851
Whether there are productive fixed assets	Invest	0.4391	0.4963	0.4084	0.4916	0.3152	0.4646
Whether to take care of grandchildren	Care	1.4797	0.4996	1.4612	0.4985	1.4335	0.6110
Logarithm of household income per capita	Income	8.4956	1.1977	7.9222	1.5583	8.0247	1.2792
**Mediating variable**							
Basic medical insurance	Insurance	0.9638	0.1866	0.9228	0.2668	0.9669	0.1787
**Observations**	–	6,752	6,752	6,752

### Empirical model

To explore the impact of health shocks on the common prosperity of rural middle-aged and elderly people, the benchmark model was constructed as follows:


(2) $$\begin{array}{l} CP_{it}=\beta_{0}+\beta_{1}X_{it}+\beta_{i}{conrtols}_{i}+\alpha_{i}+\lambda_{t}+\epsilon_{it} \end{array}$$

Where *CP* represents the common wealth level of middle-aged and elderly people; *X* stands for three variables to measure health shocks: chronic and acute health shocks as well as daily activity ability shock; *conrtols* refers to a series of selected control variables; β_0_ indicates the intercepted item; β_1_ means the coefficient of interest; β_*i*_ is the coefficient of control variables; *i* represents middle-aged and elderly individual, and *t* stands for year; ϵ_*it*_is a normally distributed random error vector. Meanwhile, variables α_*i*_ without changing with time (individual effect) and other variables λ_*t*_ changing with time (year effect) were controlled as random disturbance terms to alleviate the endogenous problem caused by missing variables.

## Empirical results and analysis

### Benchmark regression results

The regression results of the impact of three types of health shocks on the common prosperity of the middle-aged and elderly are reported in Table 3 (1), (3), and (5) are the two-way fixed effects of the control year and individual, and (2), (4), and (6) are the regression results of adding control variables on this basis. Regression coefficients shrank, but the significance and none of the coefficient directions changed. Daily activity ability and acute health shocks were significant at the 1% level, and chronic health shocks were significant at the 5% level. It indicates that health shocks significantly negatively affected the common prosperity of the rural middle-aged and elderly. The regression coefficients of the three types of health shocks are −0.0854, −0.0142, and −0.0113. It can be seen that daily activities have the largest negative effect, followed by chronic and finally acute health shocks. For every 1% increase in the impact of daily activity ability, the common wealth level of the rural middle-aged and elderly can decrease by 8.54% on average. The possible reason is that the limited daily activities of middle-aged and elderly people will increase family expenditures compared with other health shocks for one thing. For another thing, other family members are required to accompany and care for them, which not only affects the labor participation of middle-aged and elderly individuals but also restricts the labor participation of other family members. This impact may not be temporary, but long-term, which will greatly bring down the level of family economy and welfare. Besides, the regression coefficient of chronic health shocks was slightly larger than that of acute health shocks, which slightly differs from the conclusions of previous studies. This is because the number of middle-aged and elderly people with chronic diseases rather than whether they have chronic diseases was taken into consideration in this paper. Patients with chronic diseases and comorbidities in the sample occupied about 50%. The number of outpatient visits and the risk of catastrophic health expenditures will significantly increase with the increasing number of patients with comorbidities (41).

**Table 3:** The benchmark regression result.

**Variables**	**(1)**	**(2)**	**(3)**	**(4)**	**(5)**	**(6)**
ADLS	−0.0878*** (0.0107)	−0.0854*** (0.0101)				
Sick_1			−0.0183*** (0.0071)	−0.0142** (0.0063)		
Sick_2					−0.0128*** (0.0034)	−0.0113*** (0.0031)
Age		0.0145*** (0.0003)		0.0145*** (0.0003)		0.0145*** (0.0003)
Live		−0.0011 (0.0063)		−0.0016 (0.0063)		−0.0014 (0.0063)
Party		0.0014 (0.0114)		0.0012 (0.0114)		0.0016 (0.0114)
Marry		−0.0241*** (0.0089)		−0.0233*** (0.0089)		−0.0239*** (0.0088)
Hsize		0.0012** (0.0006)		0.0011* (0.0006)		0.0011** (0.0006)
Invest		0.0040** (0.0018)		0.0040** (0.0018)		0.0040** (0.0018)
Care		−0.0071*** (0.0014)		−0.0073*** (0.0014)		−0.0073*** (0.0014)
Income		0.0268*** (0.0005)		0.0268*** (0.0005)		0.0268*** (0.0005)
_cons	0.4992*** (0.0010)	−0.5562*** (0.0317)	0.4987*** (0.0013)	−0.5549*** (0.0329)	0.4982*** (0.0010)	−0.5553*** (0.0322)
Year control	Yes	Yes	Yes	Yes	Yes	Yes
Individual control	Yes	Yes	Yes	Yes	Yes	Yes
Observations	20,256	20,256	20,256	20,256	20,256	20,256

***p ≤ 0.01, **p ≤ 0.05, * *p* ≤ 0.1; Standard errors are in parentheses (the same below).

### Endogeneity and robustness test

The regression in this study made use of a two-way fixed-effects model and could alleviate the endogeneity problem that may be caused by omitted variables. However, a reverse causality existed, given that the common wealth level of middle-aged and elderly people may affect their health status in terms of the micro level. Thus, this study referred to the practice of Tian (42) and Zhang et al. (40) to select physical health status before the age of 15 and whether to exercise regularly as the instrumental variables of health shock. The instrumental variable method for testing was also applied. The regression results are presented in Table 4. The coefficients of the three core explanatory variables characterizing health shock remained significantly positive at the 1% level. It indicates the reliability of the conclusion that health shock has a significant negative effect on the common prosperity of the rural middle-aged and elderly. Meanwhile, the method of replacing explained variables was adopted to test the robustness of benchmark regression. To be specific, the three-level indicators under first-level indicators were assigned equal weights and then summed up. The latent variable was assigned 0 if ≤ 1, 1 if >1 and ≤ 2, and 2 if >2 and < 3. The panel Probit model was regressed [columns (4)–(6)], and the results all significantly supported the conclusions of this study.

**Table 4:** Results of endogenous test and robustness test.

**Variables**	**Endogenous test**			**Robustness test**		
	**(1)**	**(2)**	**(3)**	**(4)**	**(5)**	**(6)**
ADLS	−0.8447*** (0.0686)			−1.7437*** (0.1462)		
Sick_1		−0.5542*** (0.0392)			−0.2445*** (0.0742)	
Sick_2			−0.2348*** (0.0142)			−0.0966** (0.0394)
_cons	0.4058*** (0.0168)	0.3972*** (0.0192)	0.3182*** (0.0157)			
Control variables	Yes	Yes	Yes	Yes	Yes	Yes
Year control	Yes	Yes	Yes	Yes	Yes	Yes
Individual cnotrol	Yes	Yes	Yes	Yes	Yes	Yes
Observations	20,256	20,256	20,256	20,256	20,256	20,256

Due to space limitations, this study only reports the second-stage regression results. ***p ≤ 0.01, **p ≤ 0.05.

### Heterogeneity analysis

Different income groups and age structures may have different effects on common prosperity when suffering from health shocks. Therefore, heterogeneity analysis was conducted on the basis of the whole sample. The samples were classified into three groups (low-, middle- and high-income groups) according to the per capita annual income of households. Besides, age was classified into three groups (45–55, 56–64, and 65 years old and above).

The income group regression results are shown in Table 5. First, the three health shocks have the greatest impact on the common wealth level of middle-aged and elderly people among middle- and low-income groups across the board. Despite the insignificant coefficient of chronic health shocks, its sign remained negative. The impact of daily activities was taken as an example. The regression coefficients of low- and middle-income groups were −0.0969 and −0.0927, respectively, with a small difference, whose absolute value was significantly higher than that of the high-income group by about 3.1%. This indicates that low- and middle-income groups experienced a greater reduction in the level of common prosperity when suffering from health shocks compared with the high-income group. With a single source of income, low- and middle-income families mainly rely on wage income, including migrant workers, and have a relatively poor ability to resist risks. Therefore, their common wealth level drops significantly when they suffer from health shocks.

**Table 5:** Results of income groups.

**Variables**	**Low income**	**Middle income**	**High income**	**Low income**	**Middle income**	**High income**	**Low income**	**Middle income**	**High income**
	**(1)**	**(2)**	**(3)**	**(4)**	**(5)**	**(6)**	**(7)**	**(8)**	**(9)**
ADLS	−0.0927*** (0.0204)	−0.0969*** (0.0281)	−0.0659** (0.0326)						
Sick_1				−0.0206 (0.0162)	−0.0138 (0.0166)	0.0024 (0.0152)			
Sick_2							−0.0137* (0.0077)	−0.0175** (0.0085)	−0.0124* (0.0075)
_cons	−0.3346*** (0.0650)	−0.3821*** (0.0766)	0.0317 (0.0632)	−0.3357*** (0.0704)	−0.3782*** (0.0801)	0.0393 (0.0662)	−0.3253*** (0.0667)	−0.3903*** (0.0794)	0.0154 (0.0647)
Control variables	Yes	Yes	Yes	Yes	Yes	Yes	Yes	Yes	Yes
Year control	Yes	Yes	Yes	Yes	Yes	Yes	Yes	Yes	Yes
Individual control	Yes	Yes	Yes	Yes	Yes	Yes	Yes	Yes	Yes
Observations	6,749	6,386	7,121	6,749	6,386	7,121	6,749	6,386	7,121

***p ≤ 0.01, **p ≤ 0.05, * *p* ≤ 0.1.

The age group regression results are shown in Table 6. The impact of different health shocks on different age groups varied across the board. First, the regression coefficient of daily activities of the first group (45–55 years old) was significantly higher than that of the other two groups, about twice that of the other two groups. The reason for this phenomenon is that the impact on both individuals and families has a longer time span than the other two groups when 45–55-year-old individuals are limited in their ability to perform daily activities during their lifespan. For this reason, the impact on common prosperity is also greater. Secondly, acute health shocks have a greater impact on the second (56–64 years old) and third groups (65 years old and above) than the first one. Finally, the influence coefficient of chronic health shocks has a greater impact on the latter two groups despite being not significant.

**Table 6:** Results of age groups.

**Variables**	**45–55 years old**	**56–64 years old**	**>65 years old**	**45–55 years old**	**56–64 years old**	**>65 years old**	**45–55 years old**	**56–64 years old**	**>65 years old**
	**(1)**	**(2)**	**(3)**	**(4)**	**(5)**	**(6)**	**(7)**	**(8)**	**(9)**
ADLS	−0.1351*** (0.0266)	−0.0675*** (0.0204)	−0.0649*** (0.0147)						
Sick_1				0.0018 (0.0135)	−0.0139 (0.0124)	−0.0110 (0.0112)			
Sick_2							−0.0050 (0.0073)	−0.0147** (0.0065)	−0.0115** (0.0053)
_cons	−0.6450*** (0.0641)	−0.5043*** (0.0613)	−0.5594*** (0.0623)	−0.6354*** (0.0654)	−0.5066*** (0.0626)	−0.5450*** (0.0658)	−0.6422*** (0.0644)	−0.5157*** (0.0620)	−0.5545*** (0.0642)
Control variables	Yes	Yes	Yes	Yes	Yes	Yes	Yes	Yes	Yes
Year control	Yes	Yes	Yes	Yes	Yes	Yes	Yes	Yes	Yes
Individual control	Yes	Yes	Yes	Yes	Yes	Yes	Yes	Yes	Yes
Observations	6,028	7,331	6,897	6,028	7,331	6,897	6,028	7,331	6,897

***p ≤ 0.01, **p ≤ 0.05.

### Mediating effect of rural basic medical insurance

#### Results of mediating effect of basic medical insurance

To further explore whether rural basic medical insurance can alleviate the negative impact of health shocks on the common prosperity of the middle-aged and elderly, the interaction term between basic medical insurance and health shocks was introduced. If basic medical insurance can mitigate the health shocks to the common prosperity of the middle-aged and elderly, the expected interaction term coefficient was positive. The regression results are presented in Table 7. It can be seen that the interaction coefficients of the three types of health shocks and medical insurance were all positive but insignificant, indicating that basic medical insurance can alleviate the negative effect of health shocks on the common prosperity of middle-aged and elderly people, but the effect is limited. In this case, hypothesis 3 was verified. Zhang et al. (40) also maintained that basic medical insurance plays a limited role in alleviating health shocks to the decline in income and increased expenditures of middle-aged and elderly families. The possible reason lies in the focus of current rural basic medical insurance on the most basic ones, the limited reimbursement ratio, the small scope of reimbursement and the limited ability to protect vulnerable groups (43). On top of this, the gap between urban and rural medical resources is extremely large, and the construction of basic medical services in rural areas is weak. People need to seek medical treatment in other places when generally suffering from major diseases.

**Table 7:** Results of mediating effect of basic medical insurance.

**Variables**	**(1)**	**(2)**	**(3)**
ALDS	−0.0845*** (0.0101)		
ALDS*insurance	0.0141 (0.0313)		
Sick_1		−0.0142** (0.0063)	
Sick_1*insurance		0.0231 (0.0140)	
Sick_2			−0.0112*** (0.0031)
Sick_2*insurance			0.0016 (0.0079)
Insurance	0.0079** (0.0033)	0.0091*** (0.0033)	0.0088*** (0.0033)
_cons	−0.5637*** (0.0318)	−0.5633*** (0.0330)	−0.5637*** (0.0323)
Control variables	Yes	Yes	Yes
Year control	Yes	Yes	Yes
Individual control	Yes	Yes	Yes
Observations	20,256	20,256	20,256

***p ≤ 0.01, **p ≤ 0.05.

#### Benefits of rural basic medical insurance

To test the impact of rural basic medical insurance on different income groups under health shocks, all the middle-aged and elderly people participating in insurance were taken as a sample and divided into three groups according to per capita annual household income. The regression results are presented in Table 8. It was further found that low-income groups could benefit from health insurance, especially those suffering from daily activity and acute health shocks. In the regression of daily activity ability shocks, the regression coefficients of low-, middle- and high-income groups were −0.2028, −0.2445, and −0.2462, respectively. This means that medical insurance can reduce the common wealth level of the middle-aged and elderly in the low-income group by an average of 4.26% when they suffered from the impact of daily activities. In the group regression of acute health shocks, the regression coefficient of the low-income group was −0.0236, whose absolute value was significantly lower than that of middle- and high-income groups. It indicates that medical insurance can also effectively alleviate the common prosperity of the middle-aged and elderly in the low-income group in the event of acute health shocks. For chronic health shocks, the high-income group is more likely to benefit from health insurance, followed by the low-income one. Rural basic medical insurance was originally designed to alleviate the economic burden of farmers suffering from major diseases. In recent years, chronic diseases have been gradually included in the reimbursement scope. When suffering from chronic health shocks, the low-income group has less need for the utilization of medical services compared with the high-income one. In summary, the beneficiary group of rural medical insurance is the low-income group.

**Table 8:** The mediating effect of medical insurance under different income groups.

**Variables**	**Low income**	**Middle income**	**High income**	**Low income**	**Middle income**	**High income**	**Low income**	**Middle income**	**High income**
	**(1)**	**(2)**	**(3)**	**(4)**	**(5)**	**(6)**	**(7)**	**(8)**	**(9)**
ADLS	−0.2028*** (0.0129)	−0.2445*** (0.0144)	−0.2462*** (0.0171)						
Sick_1				−0.0361*** (0.0069)	−0.0378*** (0.0069)	−0.0318*** (0.0071)			
Sick_2							−0.0236*** (0.0036)	−0.0331*** (0.0038)	−0.0304*** (0.0039)
_cons	0.5801*** (0.0266)	0.2423*** (0.0395)	0.1205*** (0.0383)	0.5967*** (0.0271)	0.2540*** (0.0402)	0.1359*** (0.0387)	0.6006*** (0.0271)	0.2544*** (0.0401)	0.1416*** (0.0386)
Control variables	Yes	Yes	Yes	Yes	Yes	Yes	Yes	Yes	Yes
Year control	Yes	Yes	Yes	Yes	Yes	Yes	Yes	Yes	Yes
Individual control	Yes	Yes	Yes	Yes	Yes	Yes	Yes	Yes	Yes
Observations	6,399	6,130	6,739	6,399	6,130	6,739	6,399	6,130	6,739

***p ≤ 0.01.

## Discussion

This paper mainly aimed to construct an evaluation system for the common prosperity of rural middle-aged and elderly people under the background of the increasing aging in China. It also intends to systematically evaluate the impact of chronic and acute health shocks as well as daily activity ability shocks on their common prosperity. On this basis, whether rural basic medical insurance can alleviate the influence of health shocks on the common prosperity of the middle-aged and elderly was further explored.

Unlike absolute poverty, common prosperity is not defined by clear criteria. Under the inspiration of previous studies, a common prosperity indicator system was constructed for rural middle-aged and elderly people from material, spiritual, and social sharing dimensions, covering income, consumption, labor supply, medical resources and other factors. Different from previous studies, this paper incorporates the social sharing dimension into the indicator system and better reflects efficiency and equity. The results indicate that heterogeneity exists in the impact of health shocks on middle-aged and elderly people in rural areas. Inconsistent with other studies (40), this paper showed that daily mobility shocks have a greater impact on rural middle-aged and elderly people, followed by major disease shocks (cancer, heart disease and stroke). After a major health shock, people are inclined to perceive limited benefits. Therefore, their willingness to extend their lifespan by making a wide range of behavioral changes across the board is weakened (36, 44). In other words, the impact of a major health shock may be more significant in the short run and insignificant in the long run. However, elderly people with limited capacity for daily living need the companionship and care of other family members. In addition, they increase household expenses, which will hinder the labor participation of middle-aged and elderly people themselves and other family members of the family. This effect may be long-term rather than temporary, which will ultimately reduce household economy and welfare to a large extent. Under the common prosperity strategy, more attention should be paid to the rural middle-aged and elderly as a vulnerable group. Moreover, it is necessary to attach importance to further improvement and refinement although this study largely revealed the impact of health shocks on the common prosperity of middle-aged and elderly people in rural areas. As for measures of health shocks, mental health is also included in an increasing number of studies in addition to physical and self-rated health (45). Subsequent research can also incorporate the dimension of mental health, construct dynamic changes in health indicators, and then refine its impact on middle-aged and elderly-people in rural areas. Medical insurance has different effects on people depending on their age and income group. This study focused on middle-aged and elderly groups in the greatest need of medical services. Basic medical insurance has a limited effect on middle-aged and elderly people, which is in line with prior studies (27–30, 46). The results of this study only show this possibility without implying the failure to implement social medical insurance in China. Reasons for the results are as follows: First, more emphasis is placed on social equity in the context of common prosperity, while medical development is uneven in China, with significant differences in medical resources and coverage levels between regions. In the future, it is pressing to achieve the equalization of basic public services. Second, primary medical facilities cannot satisfy the health needs of people anymore when their age increases to a certain degree. In this case, these elderly people have no choice but to bypass primary medical institutions by going to the hospital (24). In China, however, medical treatment is low in reimbursement rates in other places or even cannot be reimbursed, which will undermine the role of medical insurance. Like a many of studies, this study used whether one participates in medical insurance, but failed to delve into the effects of different levels of medical coverage and types of insurance on middle-aged and elderly people. This is limited by data availability. The research method also focused on panel data and applied difference in difference (DID) and propensity score matching (PSM) models to explore the deeper economic relationship between the two objects (36, 47). How to integrate existing methods and conduct detailed research on medical insurance, health and common prosperity could be further investigated from multiple perspectives.

## Conclusions and implications

For the middle-aged and elderly people, disease is the biggest risk in life. This study focuses on major development needs including “Healthy China”. An indicator system was built for the common prosperity of the middle-aged and elderly in rural areas. Based on three-year tracking data, the impacts of health on the common prosperity of the middle-aged and elderly were discussed, and the effect of basic medical insurance was examined. The results show that: Frist, Health shocks hinder the improvement of and have different effects on the common prosperity of the middle-aged and seniors in rural areas. Among them, daily activity ability has the largest negative effect. Second, the impact of health shocks on different income and age groups is different. The negative effect on the common prosperity of the middle-aged and elderly in low- and middle-income groups is stronger than that in the high-income group. Daily activity ability has the greatest impact on the common prosperity of the middle-aged and elderly aged 45–55, while acute health shocks have a negative impact on the middle-aged and elderly aged above 56. Third, Rural basic medical insurance can alleviate the adverse impact of health shocks on the common prosperity of the middle-aged and elderly, but the effect is limited. In relative terms, low-income groups benefit more from it.

Based on the research conclusions, the following policy recommendations were put forward: First, the state should vigorously promote the Healthy China strategy and increase investment in the construction of rural medical infrastructure. Moreover, it shall also improve public health, and increase the breadth and depth of health service coverage. The reason is that health shocks are an important background risk in the process of promoting common prosperity. Second, the government should focus on solving the health problems of key groups including the elderly in rural areas, insist on health prevention, and regularly provide rural middle-aged and elderly people with free physical examination services. For middle-aged and elderly groups with limited daily life ability, it is necessary to increase policy preferences for them and strengthen bottom-line Protection. Third, the recommendation involves accelerating the reform of the rural basic medical insurance system, changing from an inclusive policy to a targeted one, classifying people, refining the top-level design of rural basic medical insurance, and providing more precise safeguards for vulnerable groups. In addition, the government is supposed to increase the reimbursement scope and proportion of basic medical insurance, gradually narrow the medical insurance gap between urban and rural residents and achieve the equalization of basic public services.

## Data availability statement

The original contributions presented in the study are included in the article/supplementary material, further inquiries can be directed to the corresponding authors.

## Author contributions

YZ, YS, and SC prepared the datasets. YZ performed the statistical analysis, drafted the manuscript, and contributed to study design. YS designed the study. SC, YZ, MX, and YS contributed to revision of the manuscript. MX, YZ, YS, and SC organized the study. All authors contributed to the article and approved the submitted version.

## Footnotes

^1^Notes: In 2018,1USD = 6.6118CNY; In 2015, 1USD = 6.2284CNY; In 2013, 1USD = 6.1956CNY.
